# Effects of voluntary pre-contraction of the pelvic floor muscles (the Knack) on female stress urinary incontinence—a study protocol for a RCT

**DOI:** 10.1186/s13063-021-05440-0

**Published:** 2021-07-23

**Authors:** Fátima Faní Fitz, Márcia Maria Gimenez, Letícia de Azevedo Ferreira, Mayanni Magda Perreira Matias, Maria Augusta Tezelli Bortolini, Rodrigo Aquino Castro

**Affiliations:** Department of Gynaecology, Universidade Federal de São Paulo, Rua Napoleão de Barros, 608 – Vila Clementino, CEP, São Paulo, SP 04024-002 Brazil

**Keywords:** Stress urinary incontinence, The Knack, Pelvic floor muscle training

## Abstract

**Background:**

Stress urinary incontinence (SUI) is a subtype of urinary incontinence that occurs more commonly amongst women. The pelvic floor muscle training (PFMT) is considered the gold standard for treating SUI. Another technique called the Knack postulated that pre-contraction of the pelvic floor muscles (PFM) during activities of increasing intra-abdominal pressure prevents urinary loss. Currently, there are no studies supporting the Knack for the treatment of SUI. Thus, the aim of this study is to test the hypothesis that voluntary pre-contraction of PFM can treat SUI. For this purpose, the following parameters will be analysed and compared amongst (1) the Knack, (2) PFMT and (3) the Knack + PFMT groups: urine leakage as assessed by the pad test, urinary symptoms, muscle function, quality of life, subjective cure, adherence to exercises in the outpatient setting and at home and perceived self-efficacy of PFM exercises.

**Methods:**

A single-centre, double-blind (investigator and outcome assessor) randomised controlled trial with a 3-month follow-up of supervised treatment and an additional 3 months of follow-up (unsupervised) for a total of 6 months of follow-up. Two hundred ten women with mild to moderate SUI will be included, aged between 18 and 70 years. To compare the primary and secondary outcome measures within and between the groups studied (before and after intervention), the ANOVA statistical test will be used. Primary and secondary outcome measures will be presented as mean, standard deviation, 95% confidence interval and median and minimum and maximum values.

**Discussion/significance:**

This study closes a gap, as voluntary PFM pre-contraction (the Knack) has not yet been included in the physiotherapeutic treatment of SUI, and if shown successful could be implemented in clinical practice.

**Trial registration:**

ClinicalTrials.gov NCT03722719. Registered on October 29, 2018. Study protocol version 1.

**Was this trial prospectively registered?** Yes

**Funded by**: The present study did not receive funding.

**Anticipated completion date:** The anticipated trial commencement and completion dates are October 2018 and October 2021, respectively.

**Provenance:** Not invited. Peer reviewed.

**Human research ethics approval committee:** Research Ethical Board of the Universidade Federal de São Paulo (UNIFESP), Brazil.

**Human research ethics approval number:** 2.517.312.

**Supplementary Information:**

The online version contains supplementary material available at 10.1186/s13063-021-05440-0.

## Background

Stress urinary incontinence (SUI) is defined as an involuntary loss of urine during effortful manoeuvres, such as coughing or sneezing [1] that occurs more commonly amongst women, with a prevalence of approximately 60% [2]. The severity of SUI increases with ageing; 10% of women aged 25–44 had severe incontinence compared with 15% in the age group 45–59 and 33% in the age group 60+ [1]. Urinary incontinence (UI) is a serious medical condition and a social problem because it causes shame and negative self-perception, hinders participation in social activities and impairs the psychosocial wellbeing of patients [3–5].

Conservative and surgical treatment stand out amongst the therapeutic options for SUI. The interest in a conservative approach has increased because it is less invasive and is associated with a lower risk of adverse complications compared to the high cost and risks of surgery [6–8].

Clinical practice guidelines recommend pelvic floor muscle training (PFMT) as the first-line treatment for female SUI (level A evidence) [8–10]. An alternative technique known as the Knack has also been described, which teaches patients to contract the pelvic floor muscle (PFM) before and during efforts. The Knack is a technique that proposes that the patient perform the contraction associated with her activities of daily and professional life (stress activities that increase intra-abdominal pressure, such as coughing, sneezing, laughing, physical activity) to prevent urine leakage [11]. Other studies—in which the expression “PFM functional training” was used—recommend pre-contraction (the Knack) of the PFM not only during coughing but during any activity of daily living that increases the intra-abdominal pressure [8]. The ideal urethral closure pressure to avoid urine leakage has not yet been established. Amongst continent women, PFM activation seems to be an automatic response that does not require any conscious effort [12, 13]. By learning to perform a fast, strong and timed PFM contraction, incontinent women might actively prevent the urethral descent when the intra-abdominal pressure increases [13].

The literature showed the stabilising effects of the descent of the bladder neck during the activities of increasing intra-abdominal pressure with the Knack technique. Peschers et al. evaluated nulliparous women by perineal ultrasound and electromyography during cough with and without voluntary contraction of the PFM. The descent of the bladder neck was significantly less when women were asked to contract PFM before coughing [4.7 mm (± 2.9)] than when coughing without this contraction [8.1 mm (± 2.9)]. The authors concluded that the Knack technique stabilises the bladder neck during increased abdominal pressure [12]. Miller et al. used perineal ultrasound to check bladder mobility when they coughed with and without the knack technique. The mobility of the bladder neck was significantly reduced from 5.4 to 2.9 mm when the voluntary contraction of the PFM was performed [14]. Another study investigated the immediate effect of timing a PFM contraction with the moment of leakage (the Knack) to preempt cough-related stress incontinence and confirms the effect from the Knack as immediate [15].

Currently, only one study tested the efficacy of the Knack technique for urinary incontinence. Miller et al. reported that the voluntary contraction of PFM before and during coughing reduced the urine leakage after just 1 week of training [16]. Despite being a prospective, randomised, single-blind interventional study, the study has limitations, such as the lack of sample size calculation, the intervention time of only 1 week and the fact that it directed the evaluation only to the cough event. With that, the participants were instructed to perform the pre-contraction only before and during the cough, not including other activities in which the increase in intra-abdominal pressure may occur [16]. So, the essential questions in the assessment of this technique are as follows: Does voluntary PFM pre-contraction (the Knack) prevent urine leakage during the performance of activities that increase intra-abdominal pressure? Can it be used alone for SUI treatment?

The aim of this study is to test the hypothesis that voluntary PFM pre-contraction (alone) can be a treatment for urine leakage during efforts. For this purpose, the following parameters will be analysed and compared amongst (1) the Knack, (2) PFMT and (3) the Knack + PFMT groups: urine leakage as assessed by the pad test, urinary symptoms, muscle function, quality of life, subjective cure, adherence to exercises in the outpatient setting and at home and perceived self-efficacy of PFM exercises.

### Aim and objectives

The aim of the study is to investigate the effects of voluntary PFM pre-contraction alone during activities involving an increase of the intra-abdominal pressure (the Knack) for the treatment of female SUI.

The objectives are to compare the effectiveness of the Knack alone versus PFMT alone and PFMT + the Knack for the following measures:

1. Objective cure of urine leakage after intervention, defined as leakage of < 2 g (on the 1-h pad test) immediately after the intervention and 6 months after randomisation (primary outcome measure) [17].
2. Lower urinary tract symptoms and impact of UI on the participants’ quality of life immediately after the intervention and 6 months after randomisation. These secondary outcomes will be assessed by means of the 3-day bladder diary, which considers the number of episodes of urine leakage on efforts [18]; the 1-h pad test, which evaluates the severity of urine leakage [17]; the International Consultation on Incontinence Questionnaire – Short Form (ICIQ-SF), which investigates urinary symptoms [19]; and the Incontinence Quality of Life Questionnaire (I-QoL), which is a measure of quality of life [20].
3. Self-perceived efficacy of exercises immediately after intervention and 6 months after randomisation. This parameter will be assessed by means of the Self-Efficacy Scale for Practising Pelvic Floor Exercises [21]; satisfaction and desire to perform some other treatment immediately after the intervention and 6 months after randomisation (participants will be rated “satisfied” when they do not manifest a wish to perform some other treatment and as “dissatisfied” when they do wish to perform some other treatment) [18].
4. PFM strength function as measured by digital palpation (PERFECT scheme) [22, 23], Peritron manometer [24] and pelvic floor dynamometry immediately after the intervention and 6 months after randomisation.

## Methods

### Trial design

The study will be conducted at one single centre. It will consist of a prospective, randomised and controlled clinical trial: an intervention comprising 3 months of supervised treatment and a further 3 months of unsupervised treatment for a total of 6 months of follow-up. Assessments will be performed before and after (1 to 2 weeks after the end of treatment) the intervention and 6 months after randomisation and will include structured interviews, application of questionnaires, bladder diary, pad test and physical and gynaecological examinations (Table 1).

**Table 1:** Schedule of enrolment, interventions and assessments

			STUDY PERIOD				
	Enrolment	Allocation		Post-allocation intervention delivery and research follow-up			
**TIMEPOINT**	***-t***_***1***_	**0 m**	***1-2 w***	***1 m***	***2 m***	***3 m***	***6 m***
**ENROLMENT:**							
**Eligibility screening**	X						
**Informed consent**		X					
**Allocation**		X					
**INTERVENTIONS:**							
***The knack group***			X	X	X	X	
***PFMT group***			X	X	X	X	
***The Knack + PFMT group***			X	X	X	X	
**ASSESSMENTS:**							
***Basic socio-demographic data***		X					
***BMI***		X					
***General health status Medical history and medication***		X					
***Parity and obstetric history***		X					
***Type and duration of SUI***		X					
***3-day bladder diary***		X				X	X
***1-h pad test***		X				X	X
***ICIQ-SF***		X				X	X
***PERFECT scheme***		X				X	X
***Dynamometry***		X				X	X
***Vaginal squeeze pressure***		X				X	X
***I-QoL***		X				X	X
***Frequency of the outpatient sessions***			X	X	X	X	
***Adherence to the home exercises***				X	X	X	X
***Self-efficacy and expectation***		X				X	X
***Subjective cure of SUI***						X	X
***Adverse events***			X	X	X	X	X

*PFMT* pelvic floor muscle training, *ICIQ-SF* International Consultation on Incontinence Questionnaire – Short Form, *I-QoL* Incontinence Quality of Life Questionnaire, *BMI* body mass index, *SUI* stress urinary incontinence, *m* month, *w* week

### Research site

The study will be conducted at the Urogynaecology and Vaginal Surgery outpatient clinic, Department of Gynaecology, Universidade Federal de São Paulo (UNIFESP), Brazil.

### Participants

The study population will comprise women with mild to moderate SUI or mixed urinary (MUI). The diagnosis of SUI will be based on the patient’s report of leakage during physical stress and report of the condition that bothered them the most in case of mixed urinary incontinence (stress urinary or urgency incontinence) and confirmed by means of the 1-h pad test (leakage ≥ 2 g) [17]. Leakage up to 10 g will be rated as mild SUI and 11 to 50 g leakage as moderate SUI [17]. The sample will also include women with grade 2 muscle strength (normal contraction with an elevation of the anterior vaginal wall) on the two-finger assessment rated according to the Oxford scale [22, 23].

### Recruitment

Participants will be recruited from the Urogynaecology and Vaginal Surgery outpatient clinic, Department of Gynaecology, UNIFESP. The eligibility criteria will be applied in two steps: (1) women with urinary symptoms will be assessed by a urogynaecologist, who will perform a clinical examination (clinical and physical examinations, including the cough stress test) [1] and the 1-h pad test [17]; next, the patients will be referred to the physical therapy unit, where conservative treatment will be offered; (2) a physical therapist specialised in pelvic floor rehabilitation will provide instruction on the location and function of the PFM by means of oral explanations and anatomical illustrations; teach the patients how to correctly contract the PFM checked by means of two-finger palpation (four sessions to learn how to correctly perform contractions) [25]; and invite the patients who learned how to correctly perform PFM contractions and with grade 2 muscle strength [22, 23] to participate in the study. Patients who agree to participate will be requested to sign an informed consent form (Additional file 1).

The eligibility criteria are described in Table 2. Patients who do not meet the inclusion criteria will be referred back to the physician to receive treatment for UI. Eligible patients who complete the assessment will be randomly allocated into the study groups: (1) the Knack, (2) PFMT and (3) the Knack + PFMT.

**Table 2:** Inclusion and exclusion criteria

**Inclusion criteria**	
- Age 18 to 70 years old	
- Mild to moderate SUI or MUI (with predominance of SUI) as assessed by means of the 1-h pad test (leakage ≥ 2 g). Mild SUI will be defined as leakage up to 10 g and moderate SUI as leakage of 11 to 50 g [17]	
- Able to have a gynaecological examination	
**Exclusion criteria**	
- Symptoms of overactive bladder alone	
- Chronic degenerative, uncontrolled metabolic, neurological or psychiatric diseases	
- Previous participation in a pelvic floor re-education programme and/or previous pelvic floor surgery or currently receiving other treatment for UI	
- Pelvic organ prolapse greater than stage II according to the Pelvic Organ Prolapse Quantification (POP-Q) system	
- Use of medication for UI or medication that interferes with the musculoskeletal system	
- Loss of stools or mucus	
- Active urinary or vaginal infection in the past 3 months	
- Body mass index ≥ 35 kg/m^2^	

### Interventions

The participants will perform PFM exercises for 14 weeks in individual outpatient sessions supervised by an experienced physical therapist with training in pelvic floor rehabilitation. On weeks 1 and 2, the PFM training programme will be presented and taught (two outpatient weekly sessions) as per the allocation group. From weeks 3 to 14, the participants will perform PFM strength training, power and hypertrophy. The exercise programme for the first month will begin with a single outpatient session; then, the participants will perform the exercises at home and will visit the outpatient clinic 15 days later to resolve possible doubts and train under the supervision of a physical therapist [26]. At the end of the first month of training, the participants will visit the outpatient clinic to enhance the training programme by aiming to achieve greater recruitment of the involved muscles and avoid muscle adaptation [25]. The training protocol will be divided into three stages to allow for gradual progression of treatment. This is to say, the exercises will be gradually enhanced in the terms of duration, number of repetitions and position. Each stage will last 4 weeks (Table 3).

**Table 3:** Details of the monthly training programmes

	The Knack group	PFMT group	PFMT + the Knack group
**Weeks 1–2**	Presentation and explanation of the exercise programme	Presentation and explanation of the exercise programme	Presentation and explanation of the exercise programme
**Weeks 3–6 (1st month)**	3 exercises in supine position: 8 repetitions (1 series) of each exercise 3 times per day, 3 days per week for a total of 108 monthly series + orientation to perform the Knack during ADL and WA	8 MVC of PFM in supine position sustained for 6 s (type I muscle fibres) with a resting interval twice as long between contractions, followed by 3 fast contractions (type II muscle fibres) 3 times per day, 3 days per week, for a total of 36 monthly series	8 MVC of PFM in supine position sustained for 6 s (type I muscle fibres) with a resting interval twice as long between contractions, followed by 3 fast contractions (type II muscle fibres) 3 times per day, 3 days per week, for a total of 36 monthly series 3 exercises in supine position: 8 repetitions (1 series) of each exercise 3 times per day, 3 days per week for a total of 108 monthly series + orientation to perform the Knack during ADL and WA
**Weeks 7–10 (2nd month)**	3 exercises in sitting position: 8 repetitions (1 series) of each exercise 3 times per day, 3 days per week for a total of 108 monthly series + orientation to perform the Knack during ADL and WA	8 MVC of PFM in sitting position sustained for 8 s (type I muscle fibres) with a resting interval twice as long between contractions, followed by 4 fast contractions (type II muscle fibres) 3 times per day, 3 days per week, for a total of 36 monthly series	8 MVC of PFM in sitting position sustained for 8 s (type I muscle fibres) with a resting interval twice as long between contractions, followed by 4 fast contractions (type II muscle fibres) 3 times per day, 3 days per week, for a total of 36 monthly series 3 exercises in sitting position: 8 repetitions (1 series) of each exercise 3 times per day, 3 days per week for a total of 108 monthly series + orientation to perform the Knack during ADL and WA
**Weeks 11–14 (3rd month)**	4 exercises in standing position: 8 repetitions (1 series) of each exercise 3 times per day, 3 days per week for a total of 144 monthly series + orientation to perform the Knack during ADL and WA	8 MVC of PFM in standing position sustained for 10 s (type I muscle fibres) with a resting interval twice as long between contractions, followed by 5 fast contractions (type II muscle fibres) 3 times per day, 3 days per week, for a total of 36 monthly series	8 MVC of PFM in standing position sustained for 10 s (type I muscle fibres) with a resting interval twice as long between contractions, followed by 5 fast contractions (type II muscle fibres) 3 times per day, 3 days per week, for a total of 36 monthly series 4 exercises in standing position: 8 repetitions (1 series) of each exercise 3 times per day, 3 days per week for a total of 144 monthly series + orientation to perform the Knack during ADL and WA

*MVC* maximum voluntary contraction, *s* seconds, *ADL* activities of daily living, *WA* work-related activities

Although there are no known side effects or complications from PFMT—besides possible discomfort after intervention—the occurrence of any adverse events will be monitored. All the participants will be instructed to contact the investigators in case they exhibit some adverse events at any time throughout the study.

### Outpatient sessions

The participants will perform 45-min individual PFM exercising sessions. The exercises will be performed in the supine (first month), sitting (second month) and standing (third month) positions. During the sessions, the participants will be verbally commanded to perform a series of PFM exercises under the supervision and encouragement of a physical therapist (Table 3). By the end of this 3-month training period, the participants will have attended six outpatient sessions (two per month).

### Home PFM exercises

The participants in all groups will be encouraged to perform three daily series with eight repetitions 3 days a week throughout the 3-month training period. The exercises will be performed in the supine (first month), sitting (second month) and standing (third month) positions (Table 3).

#### Group I (the Knack)

The participants will perform the Knack (Additional file 2) at the outpatient clinic and at home. The Knack consists of voluntary PFM contractions before and during activities that increase abdominal pressure. Such contraction elevates the pelvic floor cranially, with consequent closure of the urethra, vagina and rectum [11], stabilisation of the pelvic floor [12, 16] and avoidance of urine leakage [16].

Participants who are unable to perform the indicated exercises because of some physical limitation will undergo a process of adjustment according to their physical conditions. In addition to the protocol, the participants will be instructed to perform PFM contraction before and during any effort associated with daily living (ADL) and work-related (WA) activities (coughing, sneezing, laughing, walking up and down stairs, jumping, running, bending) [16].

#### Group II (PFMT)

These participants will perform PFMT at the outpatient clinic and at home. This protocol is based on studies by Bø et al. [27] (Additional file 3). The rationale underlying intensive PFM strength training is that it might develop the structural support of the pelvis by raising the levator plate to a permanent, higher position within the pelvis and promoting PFM and connective tissue hypertrophy and stiffness. These conditions facilitate automatic and more efficacious activation of motor units (neural adaptation), which impedes descent during activities that increase abdominal pressure [11].

#### Group III (the Knack + PFMT)

These participants will perform the exercises described for both the Knack and PFMT groups at the outpatient clinic and at home (Additional file 2 and 3).

### Criteria for discontinuing or modifying allocated intervention

There are no special criteria for discontinuing or modifying allocated interventions. Participants can choose to stop the treatment proposed in the study at any time before the results are published.

### Strategies to improve adherence to interventions

To motivate and improve adherence to at-home training, the participants will receive adhesive labels (of discrete colour) to place at strategic locations (computer, television set, furniture) at home or the workplace to remind them to perform the indicated exercises [28].

### Relevant concomitant care permitted or prohibited during the trial

During the protocol, medical consultations and the intake of medications are allowed, with the exception of medication or another treatment for urinary incontinence.

### Standardisation of treatment

The physical therapist who will be delivering the interventions is extensively trained in administering the standardised treatment protocols and rigorous procedures used for both the experimental and control groups. Routinely, during the course of the study, the physical therapist will meet with the study team to ensure consistency in the protocol and to discuss any concerns that may arise.

### Randomisation/blinding

#### Sequence generation and allocation concealment

Patients who agree to participate by signing an informed consent form will be stratified (to ensure sample homogeneity) into two groups based on the 1-h pad test results. The stratification will be performed according to the severity of SUI (mild leakage ≥ 2 to 10 g; moderate leakage 11 to 50 g) [17]. Next, the patients will be randomly allocated to one of three treatment groups (the Knack, PFMT and the Knack + PFMT). The allocation sequence will be generated by a research assistant (LAF) using a computer-generated random number table (http://randomization.com) [29] with a group ratio of 1:1:1 and will be concealed in sequentially numbered, sealed and opaque envelopes. The envelopes will be kept in a closed locker at the centre to which only the research assistant will have access. The envelopes will be given to the physical therapist (FFF) immediately prior to the first outpatient session.

#### Blinding

The participants will not be blinded as to the received treatment. The researchers involved in data acquisition [outcome measures (MMG)], data analysis and/or statistical analysis (professionals without knowledge of the results) will be blinded to the group allocation. The physical therapist responsible for the treatment (FFF) will not be blinded to the allocation of the patients in the groups because of the difficulties set by differences between techniques and, therefore, will not be involved in data acquisition, data analysis and/or statistical analysis. The epidemiological, clinical, allocation, randomisation and outcome data from the study will be stored in a virtual database. At the end of the study, the results will be made available to researchers who did not have contact with the participants as well as to statisticians blinded to the groups, and the researchers and statisticians will perform the data analysis.

### Outcome measures

The primary and secondary outcomes measures will be assessed by a physical therapist specialised in pelvic floor rehabilitation and the physician-investigator. The sample will be characterised based on sociodemographic [age, body mass index (BMI), duration of symptoms, number of pregnancies, number of vaginal deliveries, ethnicity and educational level)] and clinical (urinary symptoms, PFM function, quality of life) variables.

#### Primary outcome measure

The primary outcome measure will be the objective cure of UI as assessed by means of the 1-h pad test 6 months after randomisation. For the 1-h pad test, the women will be asked to wear pre-weighed pads and drink 500 ml of sodium-free liquid in < 15 min. After rest, they will be instructed to exercise for 30 min, including walking, climbing up and down five flights of stairs (10×), jumping (10×), coughing vigorously (10×), bending to pick up an object from the floor (10×) and washing hands for 1 min in running water. Before and after the test, the weight of the pad will be measured with a high-precision balance to determine the amount of leakage. For the 1-h pad test, an increase of 1 to 10 g represents mild incontinence and 11 to 50 g represents moderate incontinence. Women with urine leakage ≥ 2 g will be included because the study will be conducted in a tropical country [17]. The objective cure of urine loss after the intervention will be defined as leakage < 2 g. The pad test is considered a relevant tool for the assessment of UI treatment success and has been widely used in this type of study [30–32].

#### Secondary outcome measures

The secondary outcomes will be assessed according to the recommendations formulated by the International Continence Society [33]. The instruments with the best psychometric properties (validity, reliability and responsiveness to change) were selected for each outcome.

1. *The 3-day bladder diary:* This validated instrument assesses daytime/night-time urine leakage episodes during three consecutive days [18].
2. *The 1-h pad test:* This test assesses urine leakage in situations involving effort. For the 1-h pad test, an increase of 1 to 10 g represents mild incontinence and 11 to 50 g represents moderate incontinence [17]. The results will be rated positive when the variation in the pad weight is ≥ 2 g because the study will be conducted in a tropical country.
3. *The International Consultation on Incontinence Questionnaire – Short Form (ICIQ-SF):* A four-item questionnaire that evaluates the impact of symptoms of incontinence on quality of life and outcome of treatment (0–21 overall score, with greater values indicating increased severity) [19].
4. *The Incontinence Quality of Life Questionnaire (I-QoL):* The impact of SUI on quality of life will be assessed by the Incontinence Quality-of-Life Questionnaire (I-QoL). The I-QoL evaluates the limitations on human behaviour, psychosocial impact and social embarrassment [20].
5. *Subjective cure of SUI:* The subjective cure will be measured by asking the patients if they were “satisfied” or “dissatisfied” about their condition (urinary incontinence) after treatment. We considered them to be “satisfied” when the patient was happy with the results and did not want a different treatment and “dissatisfied” if the patient desired a treatment other than the initial one [18].
6. *Frequency of the outpatient sessions:* The frequency of the outpatient sessions will be monitored by the physiotherapist and will be expressed as a percentage of the total sessions anticipated for 3 months of supervised treatment. The participants will visit the outpatient clinic twice per month to perform training under supervision by a physical therapist. By the end of the 3-month treatment period (excluding the baseline and final assessments), the participants will have performed six intervention sessions.
7. *Adherence to home exercises:* The number of completed exercise sets will be obtained using an exercise diary, and it will be recorded as the mean of the exercise sets per month performed during the 3-month therapy intervention for both groups. The number of home exercise sets in both groups will also be assessed at the 6-month follow-up [34]. The participants must register how many times in the day they performed the indicated series of exercises (once, twice or three times per day) and the day they were performed (to calculate how many days in the month the exercises were actually performed). At the end of each month, the participants are expected to have performed the exercises for at least 12 days (3 days per week) (36 days by the end of the 3-month supervised period and 6-month follow-up). The participants will be directed to make records only when the exercises were effectively performed. The participants are expected to perform at least nine series of exercises per week for a total of 36 per month. At the end of the 3-month period of supervised training, following the primary and secondary assessments, the participants will be directed to continue the training at home (according to the protocol corresponding to the group of allocation) and will be given three diaries of exercise frequency (with the due dates) to record the exercises performed throughout the 3 months of unsupervised training at home.
8. Self-efficacy/outcome expectation to PFM exercises: The scale contains 17 questions in the format of a visual analogue scale with answers ranging from 0 (not confident at all) to 100 (the most confident). Thirteen items refer to self-efficacy and four to outcome expectations. The final scores will be obtained by calculating the mean of the items, ranging from 0 to 100, in which higher values are equivalent to more beneficial self-efficacy/outcome expectation to PFM exercises [21].
9. PFM function, strength and vaginal squeeze pressure: The aim of physical and functional pelvic floor assessment is to investigate the muscle integrity and contractile ability of the pelvic muscles [35]. All the procedures will be performed with the participants in the lithotomy position.
  - *Function of the PFM (digital palpation):* PFM function will be assessed through two-finger vaginal palpation. The examiners will place the fingers at about 4 cm of the vaginal introitus, and the muscle function will be assessed by means of the PERFECT scheme. PFM strength will be assessed by the modified Oxford Grading Scale (scores of 0–5) [22]. The volunteers will be instructed to perform three contractions lasting 5 s with the greatest strength possible (maximum PFM contractions); these periods of contraction were interrupted by 1-min rest periods. Endurance will be expressed as the length of time for up to 10 s that a maximal vaginal contraction could be sustained. Thus, the contraction will be timed until the muscle started to fatigue. The number of fast maximal PFM contractions that could be repeated (up to 10) will be reported as the fast contraction variable [23].
  - *PFM strength:* An intravaginal dynamometric speculum (Miotec®) (Porto Alegre, Rio Grande do Sul, Brasil), *New Miotool Wireless*, will be used. The vaginal dynamometer will measure resistance to unidirectional and anteroposterior compression in kilogrammes/force (kgf). The participants will be requested to perform three maximum voluntary contractions (MVCs) of the PFMs at 3-min intervals. The best of three consecutive attempts was registered as the MVC [36].
  - *Vaginal squeeze pressure:* A vaginal balloon catheter connected to a pressure transducer (Peritron manometer-Cardio DesignTM, Victoria, Australia) will be used to assess the vaginal squeeze pressure [24]. The best of three consecutive attempts will be registered as the MVC [36].

### Trial management

The principal investigator (PI) (FFF) and a research coordinator (LAF) will regularly communicate (through emails, telephone or in person) to promote and monitor the recruitment progress. The PI, the evaluators and physiotherapists will conduct conference calls or face-to-face meetings to monitor the study’s progress. All members of the research team will be informed of the progress through e-mail every 2 weeks. All the data collected will be anonymised and stored in a locked cabinet in (blinded). After each assessment and on the same day, the files will be reviewed by the research assistant to identify missing data. Any missing information will be retrieved immediately by research assistants directly from the study participants. The data will be entered weekly (depending on recruitment rate) into a computerised database, SPSS [Statistical Package for Social Sciences (SPSS Inc., Chicago, IL, EUA)], version 22.0. A final quality-control step will be performed at the time of the data analysis by the trial statistician. Frequency distributions and ranges will be analysed to detect any outliers that could signal potential errors. The data will be analysed without any nominative identifiers.

### Adverse events

In the current study, there are no anticipated risks or inconveniences, as the applied interventions and examinations are well established and widely applied in standard pelvic floor physiotherapy. All of the women will be asked during every physiotherapy and measurement consultation whether they are experiencing any adverse effects. If there are any complications or any complications are suspected, the patients will be instructed to contact a member of the study team, and they will be informed of the procedure to follow for other episodes. The Trial Steering Committee (TSC) is made up of three researchers (MMG, MMPM and RAC); they will meet weekly and analyse the ongoing results of the research. They will have the power to partially or completely halt the course of the study. No public organisations were involved in the development of this study.

### Sample size calculation

The literature on the Knack is scarce and assessed only in combination with PFMT [16, 26]. The aim of this study is to investigate the effects of the Knack alone for the treatment of SUI. Because of the lack of scientific evidence a local pilot study was previously conducted to calculate the sample size (unpublished). Forty-five patients agreeing to participate by signing an informed consent form (Additional file 1) will be recruited. This group will be randomly allocated by means of online randomisation software (http://randomization.com) to the following groups: the Knack, PFMT or PFMT + the Knack. The primary outcome measure [objective cure as established by means of the 1-h pad test (leakage ≤ 2 g)] will be used to compare the effects of SUI treatment amongst the groups. At the end of the pilot study (after the last randomised patient completes the intervention), the percentage of cured participants will be compared amongst the groups. Based on a pilot study, it is observed that the PFMT group has a higher objective cure rate compared to the knack group, however, without statistical significance at the level of 5% (*p* value = 0.592 obtained through Fisher’s exact test). Although the knack group has an objective cure rate lower than that of the PFMT group, this objective cure rate is approximately equal to 50%. The premises for the sample calculation are the hypotheses: *H*_0_: θ_1_ − θ_2_ ≤ δ and *H*_*a*_: θ_1_ − θ_2_ > δ, where, θ_1_: response rate of the Knack group; θ_2_: response rate of the PFMT group (gold standard); *δ*: Non-inferiority margin (clinically relevant difference between the response rates of groups the Knack and PFMT, and that for the calculation of non-inferiority, the value of *δ* must be negative; *r*: ratio between sample sizes *n*_2_/*n*_1_ (*r* = 1 ⇒ *n*_2_ = *n*_1_); *α*: significance level (*α* = 5%); 1 − *β*: test power (*β* = 20% ⇒ 1 − *β* = 80%) [37]. Formula:

$$ {\displaystyle \begin{array}{c}{n}_1=\frac{{\left({z}_{\alpha }+{z}_{\beta}\right)}^2\left[r{\theta}_1\left(1-{\theta}_1\right)+{\theta}_2\left(1-{\theta}_1\right)\right]}{{\left({\theta}_1-{\theta}_2-\delta \right)}^2}\\ {}{n}_2={rn}_1\end{array}} $$

Notice that, here, *r* = 1 for equal size *n*_1_ = *n*_2−_

Thus, according to the result of the pilot sample, at the 5% significance level, there is no significant difference between the groups in relation to the objective cure rate in the treatment of mild and moderate SUI, assessed by the 1-h pad test. Considering the premises: θ_1_= 42.9% and θ_2_= 71,4% (obtained through the analysis of the pilot sample), *δ* = − 7,14% (10% variation in relation to the response rate of the PFMT group, obtained through literature data [38], *r* = 1 (groups of equal sample sizes), *α* = 5% and *β* = 20% ⇒ power (1 – *β*) = 80%, we obtained a total of 122 patients (61 patients per group). Thus, considering a significance level of 5%, a sample of 122 patients (61 patients per group) has 80% power to show the non-inferiority of the knack technique compared to PFMT in the treatment of mild and moderate SUI, with a non-inferiority margin of − 7.14%, which corresponds to a variation of 10% in relation to the response rate of the PFMT group (gold standard), supposedly equal to 71.4% and assuming that the rate of response of the test group is 42.9%. The sample size calculation was based on the primary objective of comparing the Knack technique (experimental group) with PFMT (control group). However, we have a third group that associates the techniques PFMT + the knack, so, 183 participants will be recruited. It is also necessary to consider possible losses in the sample during the 6-month follow-up period, thus, we assume losses of 0 to 15% based on previously published studies [39, 40]. Thus, we will recruit nine more participants per group, increasing the total number of participants to 210.

### Statistical analysis

**Fig. 1:**
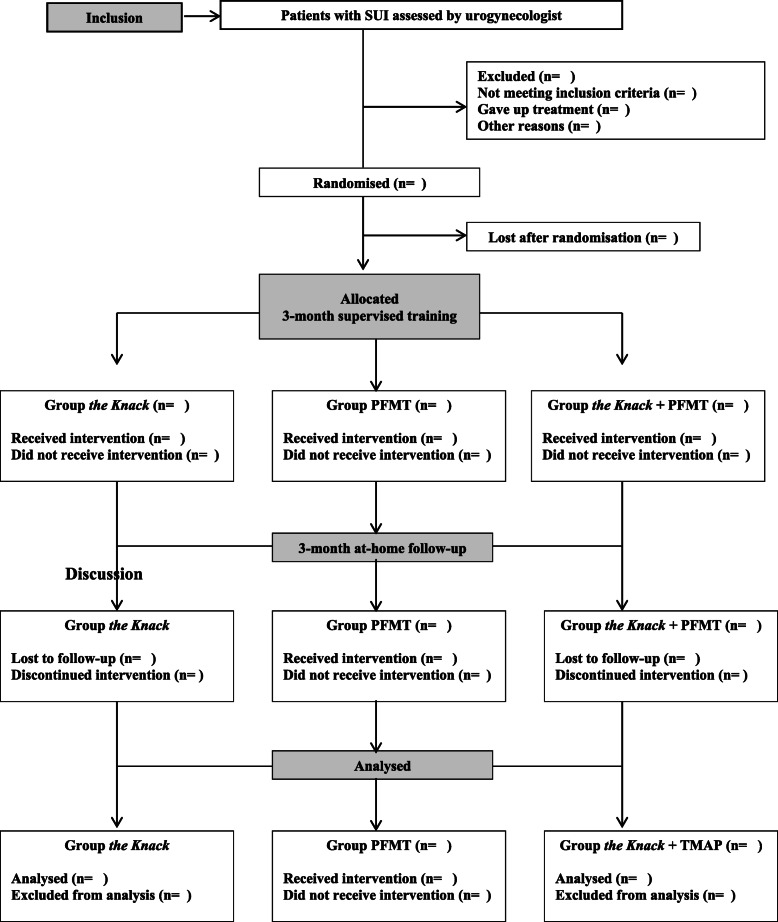
CONSORT flowchart

The statistical analysis will follow the CONSORT flowchart (Fig. 1) throughout the study stages [inclusion (assessed, excluded, randomised patients); group allocation (the Knack, PFMT, the Knack + PFMT: received or did not receive intervention), 6-month follow-up (lost to follow-up, discontinued intervention) and analysis (excluded, included patients)] [41].

Descriptive statistics will be performed for sample characterisation. Categorical variables will be expressed as the distribution of frequencies, and numerical variables by means of measures of central tendency and variability. The mean, standard deviation, 95% confidence interval, median and maximum and minimum values will be calculated for the analysis of the primary outcome measure (pad test). The primary outcome measure (pad test) will be compared within and amongst groups (the Knack, PFMT and the Knack + PFMT) (before and after intervention) using an ANOVA. All statistical analyses will be performed using the current version of SPSS software after the last patient undergoes the final assessment after intervention. The same approaches and statistical procedures used for the primary outcome measures will be employed for the analysis of the secondary outcome measures.

## Discussion

As effort activities typically provoke SUI within milliseconds [42], the focus of physiotherapy PFM training protocols on pre-contraction (the Knack) seems crucial for SUI patients. However, the gold standard SUI physiotherapy concentrates on PFMT. This study aims to distinguish each component of the pelvic floor training and to document its contribution separately; to validate Knack’s manoeuver, that is, investigating voluntary PFM pre-contractions, as a specific component of pelvic muscle training; and to determine its unique effect as a minimalist intervention.

### Dissemination of study finding

The results of this study will be disseminated through national and international scientific and professional conferences, in addition to undergraduate and postgraduate courses in PFM rehabilitation for physiotherapists.

## Trial status

This trial is actively recruiting participants (185/210). The trial is ongoing and has a planned duration of 4 years, with recruitment running from October 2018 and October 2021. If any changes need to be made to the study protocol, the relevant parts of the study will be updated, and the changes will be recorded in the clinical trials registry (ClinicalTrials.gov, NCT03722719).

## Supplementary Information

**Additional file 1.** Informed consent form.

**Additional file 2.** Details of the experimental group (The knack).

**Additional file 3.** Details of the control group (PFMT).

**Additional file 4.** SPIRIT checklist.

## Data Availability

The data analysed during the current study are available from the corresponding author upon reasonable request. The data will be available after the main manuscript is published; for other circumstances, individuals should consult the corresponding author. Any data used to support the protocol can be provided upon request.

## Abbreviations

SUI: Stress urinary incontinence
UI: Urinary incontinence
MUI: Mixed urinary incontinence
PFMT: Pelvic floor muscle training
PFM: Pelvic floor muscle
MVC: Maximum voluntary contraction
ADL: Activities of daily living
WA: Work-related activities
ANOVA: Analysis of variance
BMI: Body mass index
I-QoL: Quality-of-Life Questionnaire
ICIQ-SF: International Consultation on Incontinence Questionnaire – Short Form
SPIRIT: Recommendations for Interventional Trials
TSC: Trial Steering Committee
POP-Q: Pelvic Organ Prolapse Quantification
PI: Principal Investigator
UNIFESP: Federal University of São Paulo
