# Supplementary material for: Effects of voluntary pre-contraction of the pelvic floor muscles (the Knack) on female stress urinary incontinence—a study protocol for a RCT
Source: Trials. 2021 Jul 23;22:484. doi: 10.1186/s13063-021-05440-0 (PMC8299632; doi:10.1186/s13063-021-05440-0)
Supplement: Supplementary file 1 — Additional file 1. Informed consent form. [file 13063_2021_5440_MOESM1_ESM.docx]

**Additional file 1**

**INFORMED CONSENT FORM**

**Title: Voluntary pre-contraction of the pelvic floor muscles (the Knack) on female stress urinary incontinence – randomised controlled clinical trial**

1. You are invited to participate in a research project that seeks to establish whether contraction of the pelvic floor (vaginal) muscles performed before efforts (coughing, sneezing, bending, lifting weights, jumping, walking) might treat your urinary incontinence. Furthermore, urine leakage symptoms, vaginal muscle function, impact of symptoms on quality of life, desire to perform another treatment, frequency of exercising and your perception of the efficacy of the vaginal muscle exercises will be analysed and compared.

2. The medical and physical therapy staff that will perform the study is from the Urogynaecology unit where you are currently receiving care.

3. Throughout the study, you will undergo a consultation with an urogynaecologist physician, who will perform a gynaecological examination and investigate the presence of urine leakage.

4. Following the urogynaecology consultation, you will be referred to a physical therapist, who will perform an interview and an examination to quantify the urine leakage and assess the vaginal muscles (in the gynaecological position). You will respond to questionnaires on quality of life and perceived efficacy of exercises. Next, you will be referred for treatment by means of vaginal muscle exercises.

4.1 During the interview, your general data will be collected (name, age, marital status, occupation, educational level) and information on problems you are having because of urine leakage (assessment of quality of life and sexual function.)

4.2 Next, the vaginal muscles will be assessed in the gynaecological position. Examination will first be manual, involving touch with the fingers, while you are requested to contract the vaginal muscles. Next, the assessment will performed with a perineometer and dynamometer, which measure the muscle contraction strength. Additionally, a vaginal muscle ultrasound will be performed by a physician. None of these tests cause any pain.

4.3 Urine leakage will be assessed by means of the pad test. You will have to void your bladder and put a pad into place. Next, you will drink 500 ml of water and walk up and down the stairs, jump, bend, cough and walk for 15 minutes and wash your hands for one minute.

5. Once the assessments are over, you will be referred to one of three treatment groups. You will be randomly allocated to one such group (by means of the lottery method). Treatment will consist in vaginal muscle exercises targeting urine leakage.

- **Group 1 (previous contraction of vaginal muscles before and during efforts):** the protocol includes exercises before and after activities that increase the intra-abdominal pressure (coughing, sneezing, bending, jumping). You will have to perform an exercise protocol to train contraction before efforts and will also be oriented to perform the contractions before and during coughing, sneezing, bending to pick up something, jumping, walking, running and walking up and down stairs.
- **Group 2 (vaginal muscle training):** the treatment protocol consists of vaginal muscle training to increase their strength and promote their hypertrophy.
- **Group 3 (previous contraction of vaginal muscles before and during efforts + vaginal muscle training):** the protocol includes the exercises described for both groups 1 and 2.

5.1 The training protocol will last six months. During the first three months you must visit the Urogynaecology outpatient clinic every 15 days (twice per month) (to perform the exercises under supervision by a physical therapist) and must also perform the exercises at home. During the following three months, you will perform the exercises at home (without supervision of the physical therapy) and will return for the final assessment (described above) at the end of the six-month period.

5.2 If at the end of the study you report to be dissatisfied with the treatment performed, you will be referred for a new consultation with the urogynaecologist for assessment.

6. The risk of this treatment is very low. You might feel some discomfort during the two-finger palpation and due to the gynaecological position. Your body will be covered with a sheet until the onset of the procedures to avoid exposure.

7. The data obtained in the present study will allow for a relatively broad-scoped assessment of a new treatment for urine leakage. The resulting information will provide grounds for strategies to approach urinary incontinence better and benefit the full population of affected individuals.

8. You will not be charged for participation nor will you receive benefits such as money of gifts. However, we will reimburse any expenses you and your escort might have due to participation.

9. You are ensured comprehensive, immediate and free care for any harm resulting from participation in the study as long as necessary. You are entitled to compensation for any harm associated with the present study.

10. Your name will be hidden and stored in a confidential electronic database to protect your privacy. If you want, you may receive information on the study results.

11. You may withdraw your consent and exit the study any time you so wish. Conventional treatment will be offered to you without any hindrance.

12. Throughout the study, you will have access to the investigators to resolve eventual doubts. The principal investigator, Fátima Faní Fitz, is the physical therapist. You may also contact the Urogynaecology and Vaginal Surgery outpatient clinic at: Rua Loefgreen, 1570 – Vila Clementino, telephone +55 (11) 5576-4879; e-mail: [fanifitz@yahoo.com](mailto:fanifitz@yahoo.com). If you have any comments or doubts on the study ethics, you may contact the Research Ethics Committee (Comitê de Ética em Pesquisa - CEP) – Rua Botucatu, 572 – 1^st^ floor – cj 14, +55 (11) 5571-1062, FAX: +55 (11) 5539-7162 – E-mail: [cepunifesp@unifesp.br](file:///C:\Users\guta\Desktop\cepunifesp@unifesp.br). The CEP is composed of proficient professionals who analyse, approve and supervise studies conducted at the Federal University of São Paulo essentially as concerns their ethical aspects. **Open times (phone and in-person): Mondays, Tuesdays, Thursdays and Fridays from 9:00 to 13:00**.

**13. If you want to participate in the study, please sign this informed consent form. The form has two copies; all pages must be individually initialled and then signed at the end of the document by the study subjects or their legal representative and the principal investigator or another person(s) appointed by him/her. Each party will receive one copy of the document.**

Based on the information provided, the aims of the study entitled **“Voluntary pre-contraction of the pelvic floor muscles (the Knack) on female stress urinary incontinence – randomised controlled clinical trial”** are clear to me. I understand the procedures that will be performed, their risks and discomfort they might cause and that confidentiality and continuous availability of elucidations are ensured. It is also clear to me that my participation does not involve any expenses and that access to in-hospital care is guaranteed as per need. I voluntarily agree to participate in this study, and I will be able to withdraw consent at any point before or during the study, without any penalty, detriment or loss of any benefit I might have acquired or the care I receive at this Service.

São Paulo, ___________________________

_____________________________________

Signature of Patient / Legal Representative

_____________________________________________________________________

Signature of witness (illiterate or semi-literate volunteers or with auditory and/or visual impairments)
