# Supplementary material for: Effects of voluntary pre-contraction of the pelvic floor muscles (the Knack) on female stress urinary incontinence—a study protocol for a RCT
Source: Trials. 2021 Jul 23;22:484. doi: 10.1186/s13063-021-05440-0 (PMC8299632; doi:10.1186/s13063-021-05440-0)
Supplement: Supplementary file 2 — Additional file 2. Details of the experimental group (The knack). [file 13063_2021_5440_MOESM2_ESM.docx]

**Additional file 2**

**Training protocol Group The Knack MONTH 1**

**1. Exercise:** Trunk flexion (detaching the shoulders from the support), contracting the vaginal muscles before and during the movement.

**Initial position:** Lying down with the knees bent.

**Repetition:** 8 times.


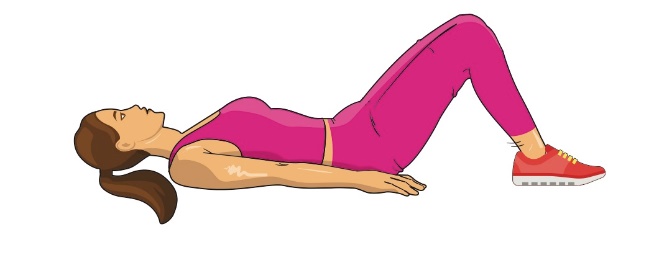


**2. Exercise:** Trunk flexion (detaching the shoulders from the support), placing the right hand on the left knee (diagonal) and contracting the vaginal muscles before and during the movement.

**Initial position:** Lying down with the knees bent.

**Repetition:** 8 times.


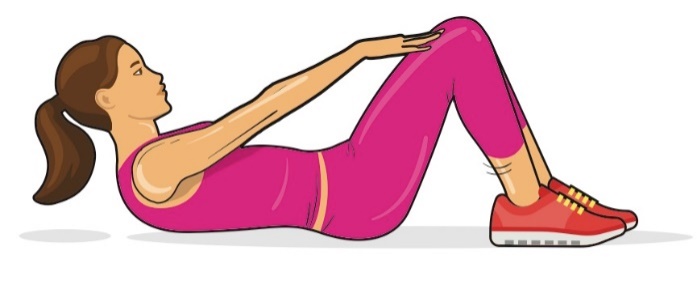


**3. Exercise:** Trunk flexion (detaching the shoulders from the support), placing the left hand on the right knee (diagonal) and contracting the vaginal muscles before and during the movement.

**Initial position:** Lying down with the knees bent.

**Repetition:** 8 times.


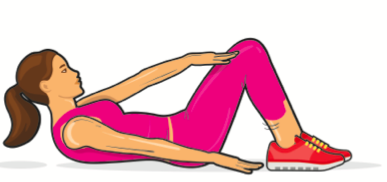


1. **Exercise:** Contract the vaginal muscles and cough once. After coughing, relax the vaginal muscles. Repeat this exercise 3 times.

**ORIENTATION**

- **PERFORM THIS SERIES OF EXERCISES 3 DAYS PER WEEK, 3 TIMES PER DAY**
- **REMEMBER TO CONTRACT THE VAGINAL MUSCLES BEFORE AND DURING ALL DAILY ACTIVITIES INVOLVING EFFORT**
- **BEFORE AND DURING COUGHING, SNEEZING, LAUGHING, WALKING UP AND DOWN STAIRS, JUMPING, RUNNING, BENDING, LIFTING A WEIGHT FROM THE FLOOR, HOLDING A CHILD, PUSHING FURNITURE**

**Training protocol Group The Knack MONTH 2**

**1. Exercise:** Rise from a seat contracting the vaginal muscles before and during the movement.

**Initial position:** Sitting with the back straight and without any support.

**Repetition:** 8 times.


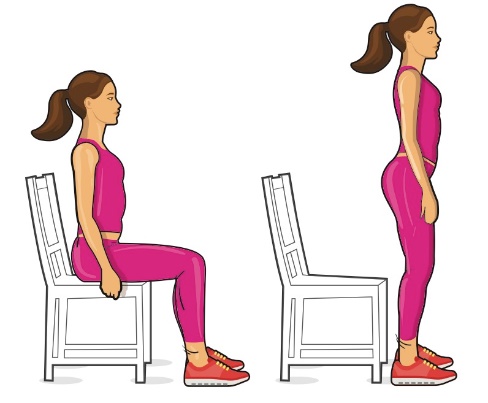


**2. Exercise:** Lift something from the floor contracting the vaginal muscles before and during the movement.

**Repetition:** 8 times.


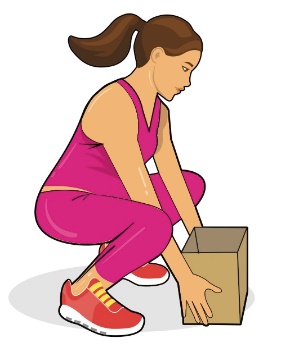


**3. Exercise:** Squat contracting the vaginal muscles before and during the movement.

**Initial position:** Standing up.

**Repetition:** 8 times.


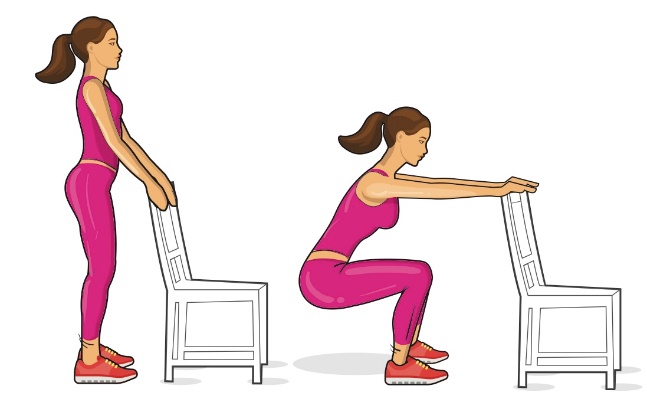


**4. Exercise:** Contract the vaginal muscles and cough twice. After coughing twice, relax the vaginal muscles. Repeat 3 times.

**ORIENTATION**

- **PERFORM THIS SERIES OF EXERCISES 3 DAYS PER WEEK, 3 TIMES PER DAY**
- **REMEMBER TO CONTRACT THE VAGINAL MUSCLES BEFORE AND DURING ALL DAILY ACTIVITIES INVOLVING EFFORT**
- **BEFORE AND DURING COUGHING, SNEEZING, LAUGHING, WALKING UP AND DOWN STAIRS, JUMPING, RUNNING, BENDING, LIFTING A WEIGHT FROM THE FLOOR, HOLDING A CHILD, PUSHING FURNITURE**

**Treatment protocol Group The Knack MONTH 3**

**1. Exercise:** Walk or run in place contracting the vaginal muscles before and during the movement.

**Initial position:** Standing up.

**Repetition:** 20 to 30 seconds.


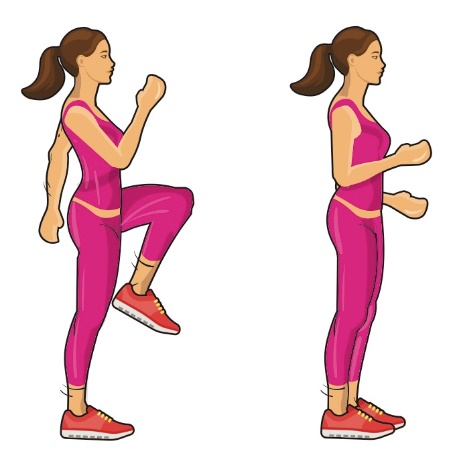


**2. Exercise:** Jump in place, contracting the vaginal muscles before and during the movement.

**Initial position:** Standing up.

**Repetition:** 8 times.


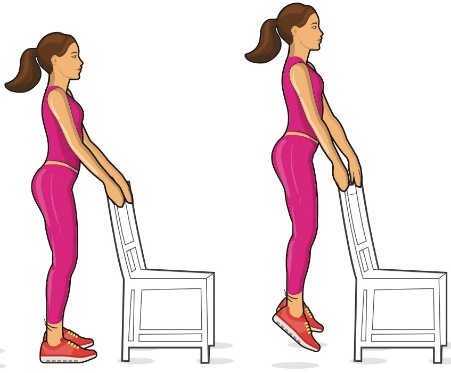


**2. Exercise:** Squat and jump in place, contracting the vaginal muscles before and during the movement.

**Initial position:** Standing up.

**Repetition:** 8 times.


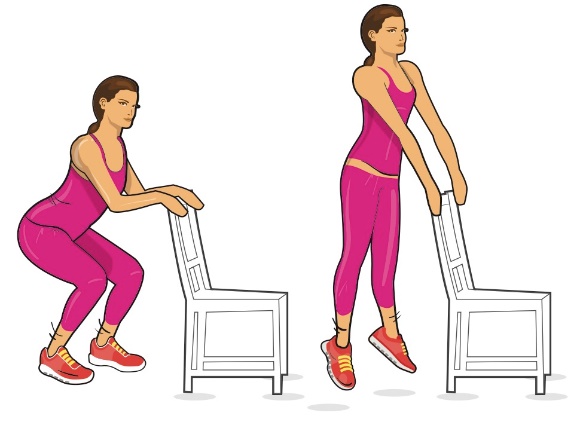


**4. Exercise:** Contract the vaginal muscles and cough 3 times. After coughing 3 times, relax the vaginal muscles. Repeat 3 times.

**ORIENTATION**

- **PERFORM THIS SERIES OF EXERCISES 3 DAYS PER WEEK, 3 TIMES PER DAY**
- **REMEMBER TO CONTRACT THE VAGINAL MUSCLES BEFORE AND DURING ALL DAILY ACTIVITIES INVOLVING EFFORT**
- **BEFORE AND DURING COUGHING, SNEEZING, LAUGHING, WALKING UP AND DOWN STAIRS, JUMPING, RUNNING, BENDING, LIFTING A WEIGHT**
