# Supplementary material for: Effects of voluntary pre-contraction of the pelvic floor muscles (the Knack) on female stress urinary incontinence—a study protocol for a RCT
Source: Trials. 2021 Jul 23;22:484. doi: 10.1186/s13063-021-05440-0 (PMC8299632; doi:10.1186/s13063-021-05440-0)
Supplement: Supplementary file 3 — Additional file 3. Details of the control group (PFMT). [file 13063_2021_5440_MOESM3_ESM.docx]

**Additional file 3**

**Illustrated exercise diary to be used by group PFMT during month 1**

**Illustrated exercise diary to be used by group PFMT during month 2**

**Illustrated exercise diary to be used by group PFMT during month 3**
